# Supplementary material for: Identification of Large Japanese field mouse Apodemus speciosus food plant resources in an industrial green space using DNA metabarcoding
Source: PLoS One. 2025 Apr 24;20(4):e0302189. doi: 10.1371/journal.pone.0302189 (PMC12021226; doi:10.1371/journal.pone.0302189)
Supplement: S1 Fig — (PDF) [file pone.0302189.s006.pdf]

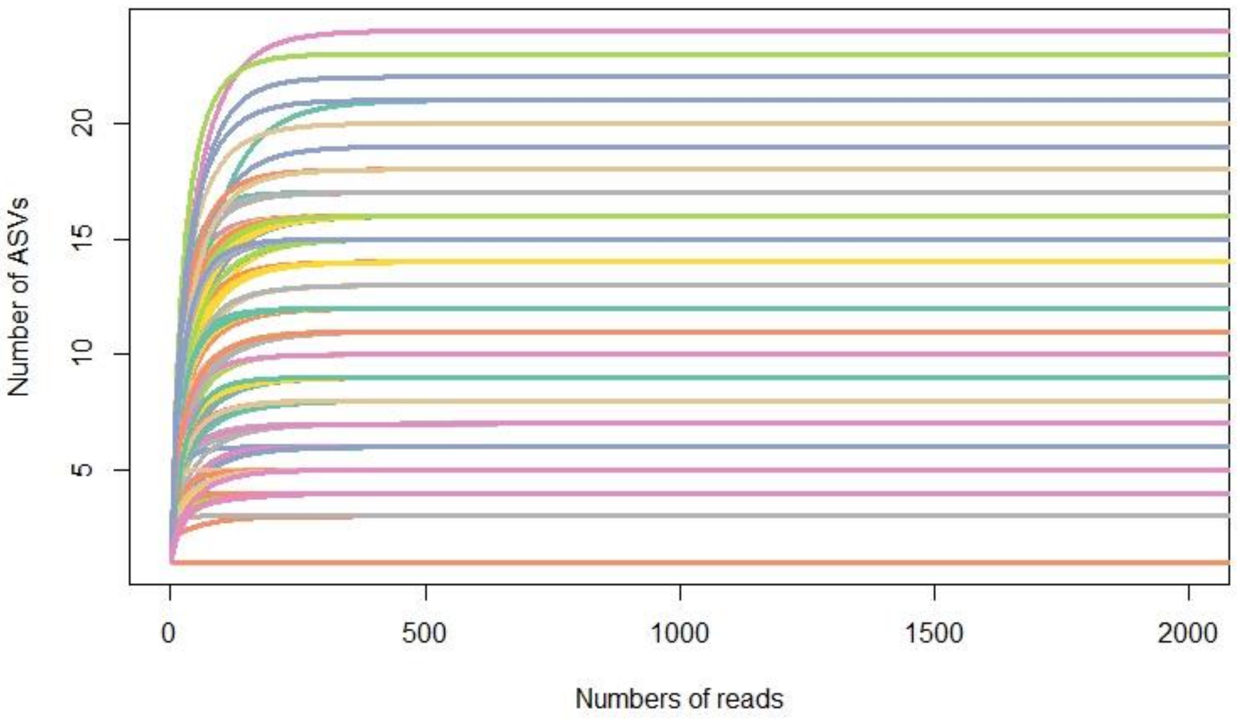

S1 Fig. The rarefaction curve was constructed using ASV data after the removal of low-frequency ASVs (1.0%).
