# Supplementary material for: Identification of Large Japanese field mouse Apodemus speciosus food plant resources in an industrial green space using DNA metabarcoding
Source: PLoS One. 2025 Apr 24;20(4):e0302189. doi: 10.1371/journal.pone.0302189 (PMC12021226; doi:10.1371/journal.pone.0302189)
Supplement: S2 Fig — The NMDS plot did not indicate seasonal differences in diet, nor did the results of the PerMANOVA (P = 0.15, F = 1.3, R2 =0.04 using the Bray-Curtis index, P = 0.07, F = 1.2, R2 =0.04 using the Jaccard index). These results were similar to the NMDS plot (Fig 3) and PerMANOVA analysis results after removing low-frequency ASVs. (PDF) [file pone.0302189.s007.pdf]

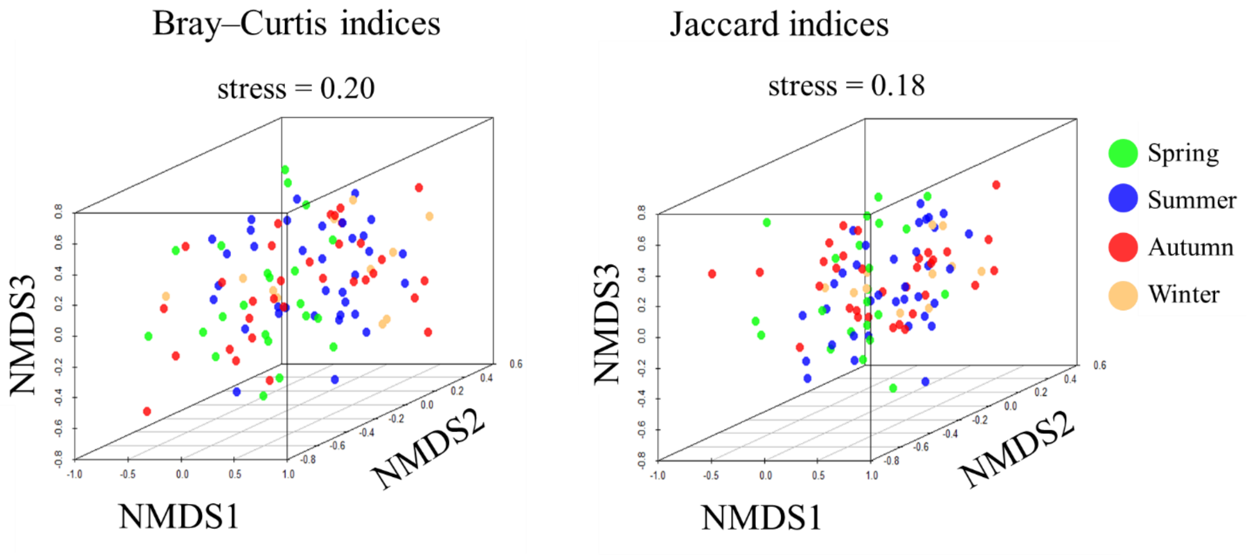

S2 Fig. Non-metric multidimensional scaling (NMDS) plot based on Bray–Curtis and Jaccard indices using all ASV information without removing low-frequency ASVs.

The NMDS plot did not indicate seasonal differences in diet, nor did the results of the PerMANOVA ($P = 0.15$, $F = 1.3$, $R2 = 0.04$ using the Bray-Curtis index, $P = 0.07$, $F = 1.2$, $R2 = 0.04$ using the Jaccard index). These results were similar to the NMDS plot (Fig 3) and PerMANOVA analysis results after removing low-frequency ASVs.
