# Supplementary material for: Identification of Large Japanese field mouse Apodemus speciosus food plant resources in an industrial green space using DNA metabarcoding
Source: PLoS One. 2025 Apr 24;20(4):e0302189. doi: 10.1371/journal.pone.0302189 (PMC12021226; doi:10.1371/journal.pone.0302189)
Supplement: S3 Fig — These maps were draw used JAXA High Resolution Land-Use and Land-Cover Map of Japan [68]. (A) ▲ indicate Forest Research Station, the Field Science Center for Northern Biosphere, Hokkaido University. (B) Sato et al. 2019 [38] was conducted survey on Ikuchijima, Hakatajima, Kamikamagarijima, Ohmishima, Ohsakishimojima, Ohshima and Shimokamagarijima. Sato et al. 2022 [39] was conducted survey on Innoshima. (C) Dotted lines indicate areas where the Large Japanese field mice (Apodemus speciosus) were trapped. Republished from Homepage of High-Resolution Land Use and Land Cover Map Products (https://earth.jaxa.jp/en/data/2562/index.html) under a CC BY license, with permission from Japan Aerospace Exploration Agency, original copyright 3 Feb. 2025. (DOCX) [file pone.0302189.s008.docx]

A) Map around the study area in Sato et al. 2018 [37]


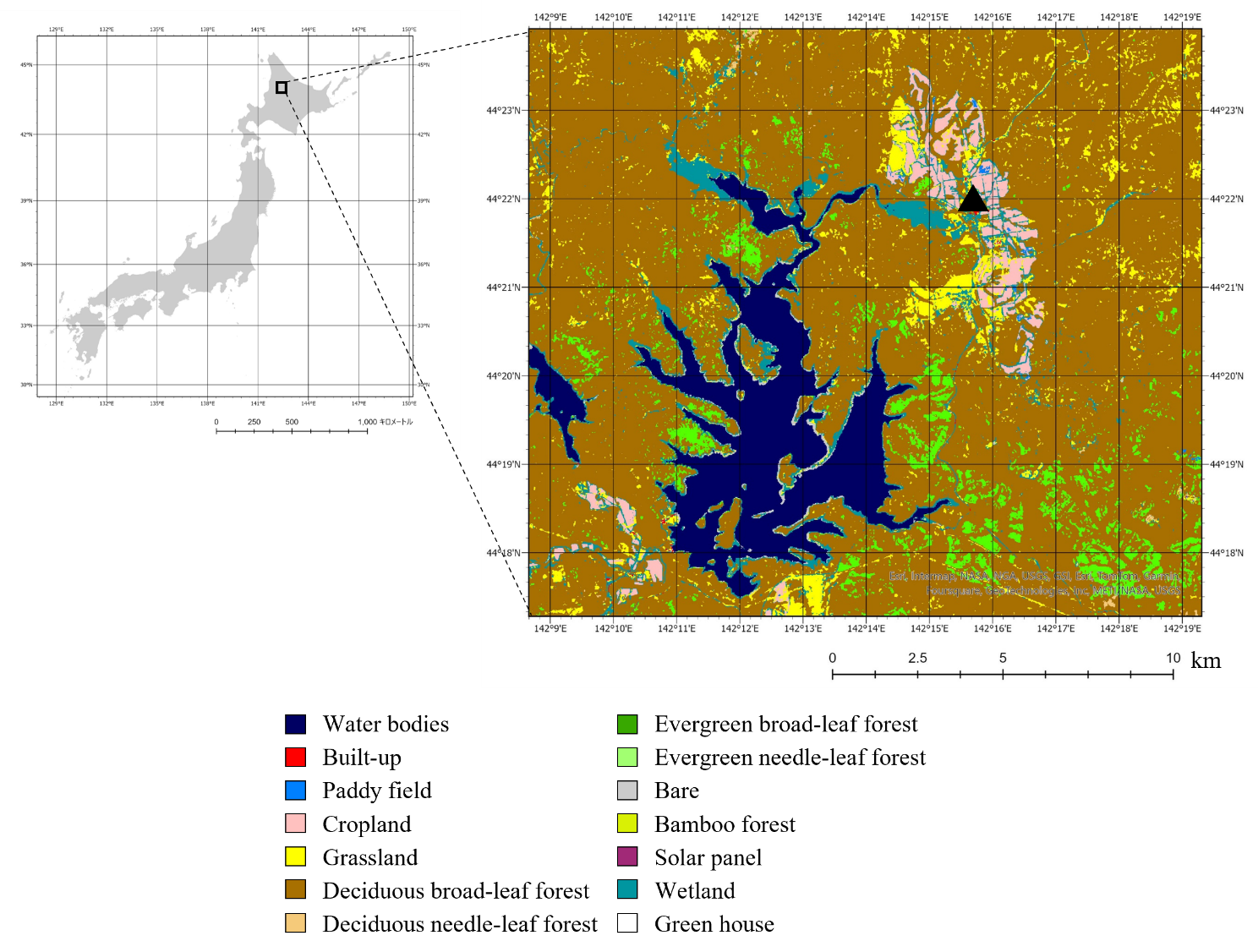


km

B) Map around the study areas in Sato et al. 2019 and 2022 [38, 39]


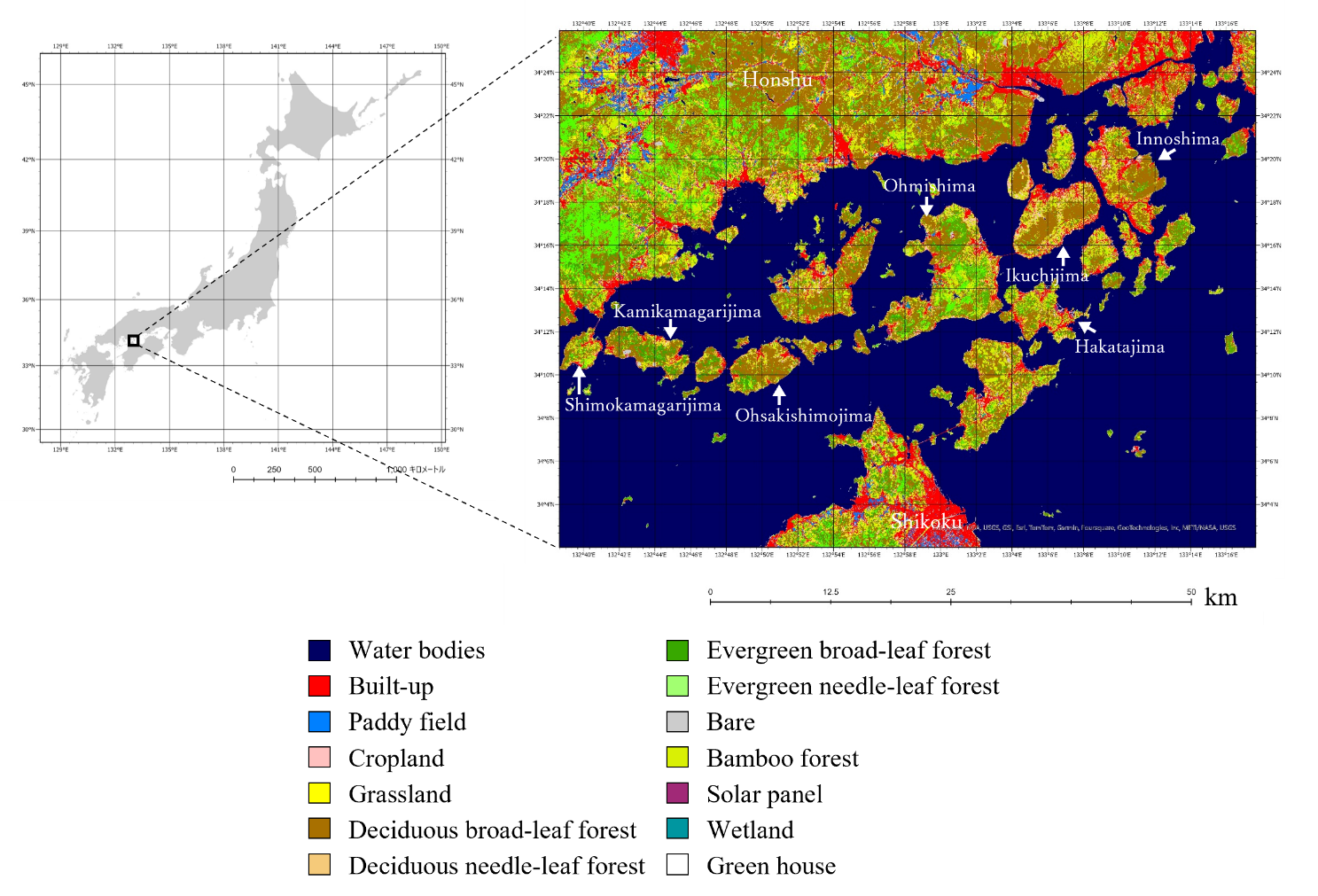


km

C) Map around this study area


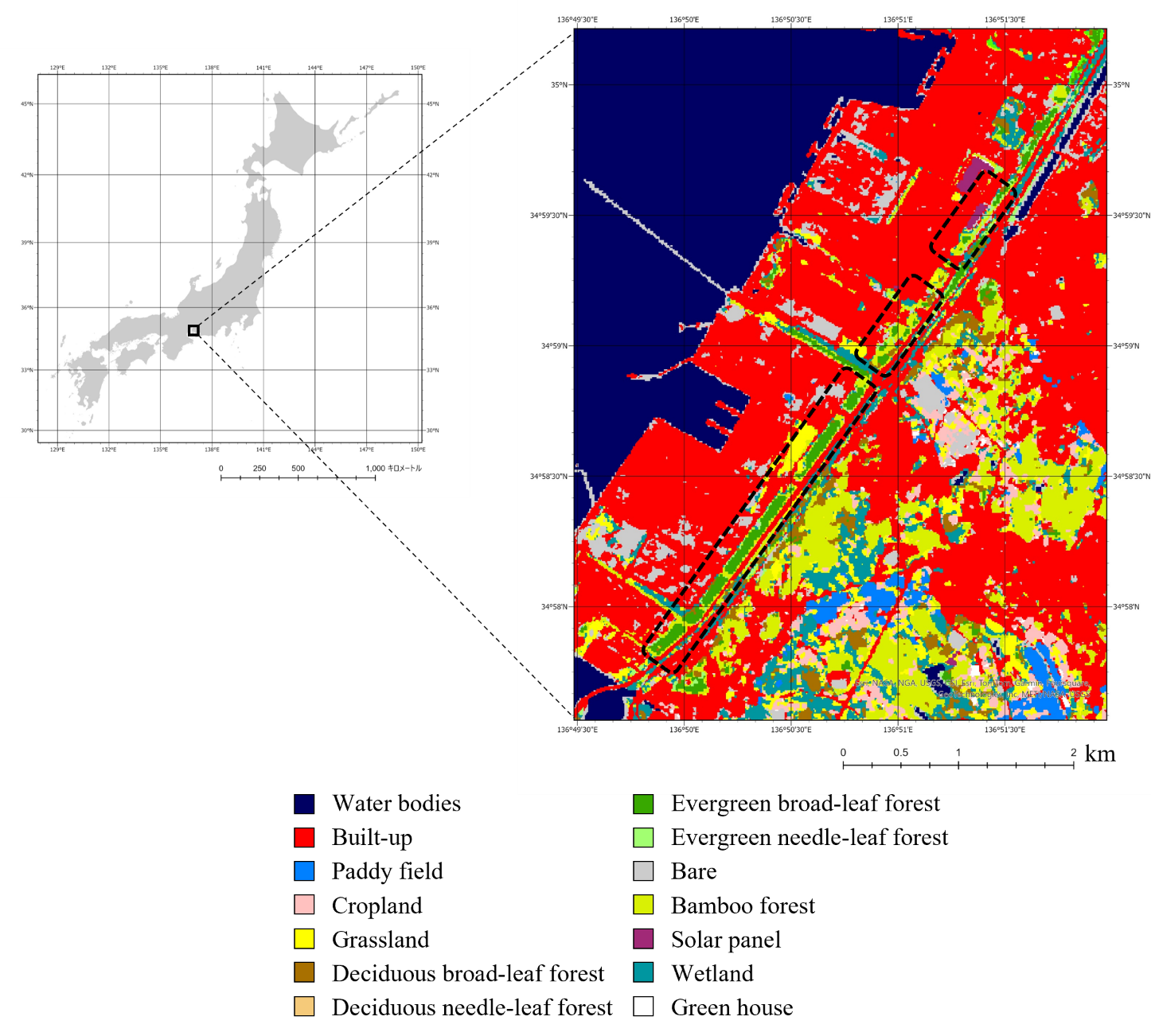


km

**S3 Fig. The Land-Cover Map of the area surrounding the in the previous studies and this study of the food plant resources utilized by the Large Japanese field mice (*Apodemus speciosus*) using DNA metabarcoding.**

These maps were draw used JAXA High Resolution Land-Use and Land-Cover Map of Japan [68]. (A) ▲ indicate Forest Research Station, the Field Science Center for Northern Biosphere, Hokkaido University. (B) Sato et al. 2019 [38] was conducted survey on Ikuchijima, Hakatajima, Kamikamagarijima, Ohmishima, Ohsakishimojima, Ohshima and Shimokamagarijima. Sato et al. 2022 [39] was conducted survey on Innoshima. (C) Dotted lines indicate areas where the Large Japanese field mice (*Apodemus speciosus*) were trapped. Republished from Homepage of High-Resolution Land Use and Land Cover Map Products (https://earth.jaxa.jp/en/data/2562/index.html) under a CC BY license, with permission from Japan Aerospace Exploration Agency, original copyright 3 Feb. 2025.
