# Supplementary material for: Identification of Large Japanese field mouse Apodemus speciosus food plant resources in an industrial green space using DNA metabarcoding
Source: PLoS One. 2025 Apr 24;20(4):e0302189. doi: 10.1371/journal.pone.0302189 (PMC12021226; doi:10.1371/journal.pone.0302189)
Supplement: Attachment — Submitted filename: Response to Reviewers.docx [file pone.0302189.s011.docx]

Dear Editor,

Thank you for your useful comments. The manuscript has been revised according to the reviewers' comments.

Dear Reviewer #1

Thank you for your useful comments. Responses to your comments are given below.

**Reviewer comment R1-1:**

I still cannot understand what the factor affecting the difference between this study and previous studies regarding the seasonal dietary changes is. Your should discuss more about the reason why you did not detect remarkable seasonal changes in diets compared with the previous studies.

**Response:**

Revised manuscript: L

We have added a discussion and Support_Figure1 regarding the vegetation information surrounding each study site.

Further discussion was considered inconclusive without a detailed comparison of vegetation and carries the risk of over-discussion.

**Reviewer comment R1-2:**

P2L29: feces sample > fecal sample

**Response:**

Revised manuscript: L29

We have corrected this sentence according to the Editors’ comment.

**Reviewer comment R1-3:**

P2L37-38: It would be more important to show that your vegetation survey and your sequencing the rbcL gene for 104 dominant plant species were performed to construct a local plant database including 651 plant species of the 796 plants growing in the study site.

**Response:**

Revised manuscript: L36-39

We have corrected this sentence according to the Editors’ comment.

**Reviewer comment R1-4:**

P4L74 and P4L80: [23] would not be an appropriate reference which is based on the tundra ecosystem and not related to A. speciosus.

**Response:**

Revised manuscript: L73-74, 81

We have corrected the reference and made corresponding adjustments to the main text according to the Editors’ comment.

**Reviewer comment R1-5:**

P4L84: contribute > provide

**Response:**

Revised manuscript: L85

We have corrected this sentence according to the Editors’ comment.

**Reviewer comment R1-6:**

P5L97-97: food resources > food plant resources

**Response:**

Revised manuscript: L98-99

We have corrected this sentence according to the Editors’ comment.

**Reviewer comment R1-7:**

P5L110-111: I still have concerns on the reason why you did not use ITS regions for this study. You explained that the ITS has not been widely applied. However, there have been 27 dietary DNA metabarcoding studies for rodents using the plant markers to date. Among them, 12 papers used ITS2, 19 papers used trnL, and only 2 papers used rbcL marker. Especially in Japan, Murano et al. (2023; https://doi.org/10.3106/ms2023-0015), Sato et al. (2023; https://doi.org/10.3106/ms2023-0003), and Kobayashi et al. (2024; https://doi.org/10.3106/ms2024-0007) used the ITS marker for several rodent species. What you explained here is not consistent with the rodent DNA metabarcoding studies. You should describe the current situation more correctly, citing these relevant papers.

**Response:**

Revised manuscript: L110-126

We think no reason to limit the DNA region of analysis of DNA barcoding to rodent food analysis methods.

As reason for DNA barcoding region selection, we focused on the 796 species found in the study area and searched the number of registrations to NCBI in the rbcL, ITS2 and trnL regions using NCBI web site. Taxonomy ID and analysis DNA region name were used for the search.

Search method:

Search the following keywords at NCBI

txidXXXX[Organism:exp] AND YYYY[All Fields]

XXXX: taxonomi ID

YYYY: DNA region name (rbcL, transcribed+spacer+2, tRNA-Leu)

The results of this analysis showed that the number of registrations for rbcL was the highest. This result was added to the method section to reinforce the justification for using rbcL region.

**Reviewer comment R1-8:**

P6L125: Fig. 1 > Add longitude and latitude in the left figure.

**Response:**

Revised manuscript: Fig. 1

We have corrected these references according to the Editors’ comment.

**Reviewer comment R1-9:**

P7L149: feces sample > fecal sample

**Response:**

Revised manuscript: L159

We have corrected these references according to the Editors’ comment.

**Reviewer comment R1-10:**

P7L150-151: How long it takes between your sampling in the field and preservation in the freezer in your laboratory and how (under what temperature) you kept your sample in 1.5 ml tube during the transportation should be described.

**Response:**

Revised manuscript: L160-162

Samples were stored at room temperature (3-5 hours) from the time of collection until stored in the laboratory freezer.

We have corrected this sentence according to the Editors’ comment.

**Reviewer comment R1-11:**

P8L168-169: Probably in the iSeq platform, the adjacent sequencing position would be separated, so that this problem is not likely to occur. Not all the Illumina sequencing platform are caused by this problem. Please check it and be careful of the expression.

**Response:**

We have received a response from Illumina's official support stating that, even for patterned flow cells on the iSeq, considerations regarding base diversity should be taken into account.

Please also refer to the following URL.

https://knowledge.illumina.com/instrumentation/general/instrumentation-general-reference_material-list/000001527

Improving nucleotide diversity is necessary even for iSeq.

**Reviewer comment R1-12:**

P11L222: What number was the minimum read number among samples to determine the number 1000 for analyses? Please note it if possible.

**Response:**

Revised manuscript: L233

We added Column (Low frequency removal reads) to Support_Table4 and Support_Figure2 in this sentence according to the Editors’ comment.

**Reviewer comment R1-13:**

P13L254: feces sample > fecal sample

**Response:**

Revised manuscript: L265

We have corrected this sentence according to the Editors’ comment.

**Reviewer comment R1-14:**

P14L268: feces sample > fecal sample

**Response:**

Revised manuscript: L279

We have corrected this sentence according to the Editors’ comment.

**Reviewer comment R1-15:**

P15L296: > > relative read abundance

**Response:**

Revised manuscript: L307

We have corrected this sentence according to the Editors’ comment.

**Reviewer comment R1-16:**

P17L325: > to bait or plants growing at the study site.

**Response:**

Revised manuscript: L336

We have corrected this sentence according to the Editors’ comment.

**Reviewer comment R1-17:**

P18L350: Is “98% percent identity” a correct expression? Probably, “percent” would not be necessary.

**Response:**

Revised manuscript: L362

We have corrected this sentence according to the Editors’ comment.

**Reviewer comment R1-18:**

P19L358: across of? Write correct English.

**Response:**

Revised manuscript: L3370

We have corrected this sentence according to the Editors’ comment.

**Reviewer comment R1-19:**

P19L362: Note here that Quercus is a member of Fagaceae.

**Response:**

Revised manuscript: L3372

The details of Fagaceae-1 and Quercus are provided in Table 1.

The main text has been corrected to clarify that these are identified taxon names in this study.

**Reviewer comment R1-20:**

P19L367-369: How about the result if you separate winter and the other seasons? Fig. 4 and 5 showed the difference between them.

**Response:**

The rationale for comparing winter and other seasons is unclear, and there is also a bias due to the uneven number of fecal samples collected. Therefore, this additional analysis was not performed.

**Reviewer comment R1-21:**

P19L372: generated compare? Write correct English.

**Response:**

Revised manuscript: L385

We have corrected this sentence according to the Editors’ comment.

**Reviewer comment R1-22:**

P28L423: [37] was a report from June to September in Hokkaido, [38] was a report from February to May in the Seto Inland Sea, and [39] was a report from March to August in the Seto Inland Sea. Information here should be wrong.

**Response:**

Revised manuscript: L434-442

We have corrected this sentence according to the Editors’ comment.

**Reviewer comment R1-23:**

P28L435-P29437: “the number of fecal samples is small” cannot be a reason for excluding this study for comparison. Instead, small numbers would be related to fewer detection of plant taxa. This is a point that should be discussed.

**Response:**

This comparison is based on the number of detections plant taxa per fecal sample, so there is no correlation between the number of analyzed fecal samples and the detection count.

However, "Sato et al. [37]" refers to a cumulative sample size of 12, which is limited compared to other studies. Considering the variability in the number of detected plant species per fecal sample, it was deemed better to exclude it from the analysis.

**Reviewer comment R1-24:**

P2pL439: 60 mg is so specific value. Please show the data to support this statement.

**Response:**

There is no clear basis for the 60 mg. We determined that 60 mg is appropriate based on the fecal weight collected in this study.

**Reviewer comment R1-25:**

P29L446: feces sample > fecal sample

**Response:**

Revised manuscript: L461

We have corrected this sentence according to the Editors’ comment.

**Reviewer comment R1-26:**

P30L459-461: I cannot understand why vegetation environment was not favorable for A. speciosus despite of the presence of more than 700 plant species. What may be the reason?

**Response:**

Revised manuscript: L485-490

In this study area, about 700 species have been identified, however the primary plants are the 104 species we collected. To clarify the causes of the foraging characteristics of winter in this study, detailed vegetation survey results, including those from previously reported study sites [37, 38, 39], are necessary. Therefore, with the current information, further discussion would result in overinterpretation, so we have limited our description to the facts derived from the results.

**Reviewer comment R1-27:**

P31L486: Discussion > discussion

**Response:**

Revised manuscript: L506

We have corrected this sentence according to the Editors’ comment.

**Reviewer comment R1-28:**

P32L499: What is “seasonal forage plants”? Are they plants that A. speciosus could eat in all the season? Or combination of various kinds of plants that were consumed by A. speciosus in different seasons?

**Response:**

Revised manuscript: L519

We have corrected this sentence according to the Editors’ comment.

Dear Reviewer #2

Thank you for your useful comments. Responses to your comments are given below.

**Reviewer comment R2-1:**

General: When referring to ITS, refer specifically to ITS2 throughout.

**Response:**

Revised manuscript: L104, 107, 120, 121, 124

We corrected from "food plant preference" to "food plant resources".

**Reviewer comment R2-2:**

Lines 108-109: Regarding increased cost of multiple markers, the authors should address that multiplex PCR protocols exist for co-amplification of rbcL and ITS2. This allows both markers to be processed for the same price as one. Here is a paper that describes this:

Sanchez DE, Dikeman AL, Lyman JA, Zahratka J, Fofanov V, Walker FM, Chambers CL. 2024. Forbs, graminoids, and lepidopterans: breadth and seasonal variation in the diet of the New Mexico jumping mouse (Zapus luteus). Journal of Mammalogy. 105(4):880–898. doi:10.1093/jmammal/gyae026.

**Response:**

When conducting DNA metabarcoding, we outsource the task to IDEA Corporation. Indeed, it is possible to analyze multiple regions in a single run using next-generation sequencers. However, since our laboratory outsources the work, costs are incurred depending on the number of samples.

We hope for your understanding.

**Reviewer comment R2-3:**

Lines 212-213: Non-dietary taxa can still occur at levels above this threshold, as Ando et al. (2018) also discuss. Newer science shows that these thresholds can strongly affect diversity statistics and so I recommend the authors repeat their statistical analysis of ASVs without a read threshold to determine if the threshold could be artificially influencing the results. In response to the authors’ rebuttal, I am not suggesting that the threshold be taken out but that it be validated to ensure the threshold is not affecting statistical results. This additional analysis can be supplementary material and will add sufficient statistical rigor to this work. I provide reference for further information regarding the effects on diversity statistics.

**Response:**

Revised manuscript: L381

And, we have added the NMDS analysis results utilizing all ASV information as a supporting figure2.

We understand that changes in the threshold affect alpha and beta diversity. However, we recognize that this issue is still under discussion（Tercel et al. 2022, doi: 10.1111/mec.16576）.

In this study, we did not include negative controls. Therefore, as noted in the text, contamination is present. No significant differences were observed in the beta diversity analysis (NDMS and PerMANOVA) with or without ASV filtering (Fig. 3 and Supporting Figure 2). When compared threshold-based filtering and no filtering, we consider threshold-based filtering to be appropriate for this study.

**Reviewer comment R2-4:**

Lines 283-285: Please use precise terminology: “frequency of occurrence” or “proportion/percent frequency of occurrence”. See the citation below:

**Response:**

Revised manuscript: L294

We have corrected this sentence according to the Editors’ comment.

**Reviewer comment R2-5:**

Lines 285-287: This sentence is still difficult to understand. I recommend saying “We summarized taxonomic resolution of each ASV/taxon by …”, rather than describing “accuracy”.

**Response:**

Revised manuscript:L〇-〇

We have corrected this sentence according to the Editors’ comment.

**Reviewer comment R2-6:**

Line 309: Is this meant to be alpha = 0.05? Otherwise, the results of this statistical analysis should be in the results.

**Response:**

Revised manuscript: L312, 320-321

We have corrected this sentence according to the Editors’ comment.

**Reviewer comment R2-7:**

Line 368: Report the actual P-values.

**Response:**

Revised manuscript: L379-380

We have corrected this sentence according to the Editors’ comment.

**Reviewer comment R2-8:**

Line 505: Identifying taxa occurring in negative controls does not necessarily mean that those sequences need to be eliminated from the dataset. If a high concentration or high frequency diet item contaminates another sample, it might not be wise to omit it.

**Response:**

Revised manuscript: L525

We have corrected this sentence according to the Editors’ comment.
