# Supplementary material for: Identification of Large Japanese field mouse Apodemus speciosus food plant resources in an industrial green space using DNA metabarcoding
Source: PLoS One. 2025 Apr 24;20(4):e0302189. doi: 10.1371/journal.pone.0302189 (PMC12021226; doi:10.1371/journal.pone.0302189)
Supplement: Attachment — Submitted filename: Response_to_Reviewers_auresp_3.docx [file pone.0302189.s012.docx]

Dear Editor,

Thank you for your valuable comments. We have revised the manuscript in accordance with the reviewers' and editorial feedback

Dear Editor

Thank you for your useful comments. Responses to your comments are given below.

**Editor comment E1:**

Furthermore, while the authors revised lines 37–38 based on Reviewer 1's comments, I disagree with the change. I believe the original sentence provided by the authors better emphasized the importance of creating a local database. Including the number of sequences in the database detracts from the broader significance of this statement.

**Response:**

Revised manuscript: L36-39

We have corrected this sentence according to the Editors’ comment.

**Editor comment E2:**

Line 134: consists of -> is located in

**Response:**

Revised manuscript: L134

We have corrected this sentence according to the Editors’ comment.

**Editor** **comment E3:**

Line 135 - 140: Required English edition for clarity

**Response:**

Revised manuscript: L137-139, L144

This survey site is owned by some corporations, and there are no publications available from which vegetation information can be referenced. The vegetation survey results we have obtained represent the most detailed data available (see S1 Table).

The correction of this comment corrects Support Table 3 to 1, Support Table 1 to 2, and Support Table 2 to 3.

**Editor comment E4:**

Line: 148: suffering -> animal suffering

**Response:**

Revised manuscript: L149

We have corrected this sentence according to the Editors’ comment.

**Editor comment E5:**

Line 150: and -> with

**Response:**

Revised manuscript: L152

We have corrected this sentence according to the Editors’ comment.

**Editor comment E6:**

Line 159 -> minimize

**Response:**

Revised manuscript: L160

We have corrected this sentence according to the Editors’ comment.

**Editor comment E7:**

Line 254: DNA databank -> database

**Response:**

Revised manuscript: L256

We have corrected this sentence according to the Editors’ comment.

**Editor comment E8:**

Line 254-355: and that this -> which

**Response:**

Revised manuscript: L256

We have corrected this sentence according to the Editors’ comment.

**Editor comment E9:**

Line 298: by -> relative to

**Response:**

Revised manuscript: L299

We have corrected this sentence according to the Editors’ comment.

**Editor comment E10:**

Line 374: after -> following

**Response:**

Revised manuscript: L376

We have corrected this sentence according to the Editors’ comment.

**Editor comment E11:**

Line 374: are identifiers used to -> serve as identifiers to

**Response:**

Revised manuscript: L376

We have corrected this sentence according to the Editors’ comment.

**Editor comment E12:**

Line 392 - 393: the statement is correct however you should mention that the number of winter samples was limited

**Response:**

Revised manuscript: L393-394, L475

We have corrected this sentence according to the Editors’ comment.

Dear Reviewer #1

Thank you for your useful comments. Responses to your comments are given below.

**Reviewer comment R1-1:**

Fig. 1: Exclude “include” in parentheses.

**Response:**

Fig 1:

We have corrected this sentence according to the Reviewers’ comment.

**Reviewer comment R1-2**

L446: analyzed region > analyzed DNA region

**Response:**

Revised manuscript: L449

We have corrected this sentence according to the Reviewers’ comment.

**Reviewer comment R1-3**

L446-447: There is a paper which showed the opposite trend: Mallot et el. (2018) https://doi.org/10.1371/journal.pone.0199556. This paper showed superiority of trnL over rbcL. It is not fair to show only one side view.

**Response:**

Revised manuscript: L449-450

We have corrected this sentence according to the Reviewers’ comment.

**Reviewer comment R1-4**

L456-458: This sentence is vague and therefore should be excluded because we cannot know the minimum quantity. The maximum volume of feces is limited by the DNA extraction kit (or method). It has been indicated that biological replication increased the obtained dietary taxa (Mata et al. 2019). That means that there we cannot estimate the total dietary taxa with feces less than 200 mg (usual maximum weight in the DNA extraction kit) and we need to apply biological replication with cost to know overall dietary trend.

Mata, V. A., Rebelo, H., Amorim, F., McCracken, G. F., Jarman, S. and Beja, P. 2019 How much is enough? Effects of technical and biological replication on metabarcoding dietary analysis. Molecular Ecology 28: 165–175.

**Response:**

Revised manuscript: L461

We understand that the required weight of feces varies not only depending on the DNA extraction kit but also due to uncontrollable factors such as microbial content and the feeding amount. However, in the case of the analysis of *Apodemus speciosus*, it is true that there is variability in the fecal weight currently used for analysis. No studies on dietary resource analysis of this species have focused on fecal weight, and it is considered an important finding suggested by this research. Therefore, we would like to retain the expression as it is here.

This study：feces (ca. 60 mg)

Shirako et al. 2014: feces (50~500 mg)

Shirako et al. 2015: gastric contents (ca. 25–75 mg)

Sato et al. 2018, 2019: feces (only three fecal pellets)

However, since it is impossible to know the "minimum quantity" of feces required for the analysis, we modified it to "optimal amount".

**Reviewer comment R1-5**

L523-533: You can refer to Sato (2024) for the issue of contamination. If the review below is useful for your explanation, cite it because you did not cite any literature in this paragraph (therefore, it is not clear how the contamination issue is problematic).

**Response:**

Revised manuscript: L538-544

We have corrected this sentence according to the Reviewers’ comment.

And we added some references.
